# Distinct Merkel Cell Polyomavirus Molecular Features in Tumour and Non Tumour Specimens from Patients with Merkel Cell Carcinoma

**DOI:** 10.1371/journal.ppat.1001076

**Published:** 2010-08-26

**Authors:** Hélène C. Laude, Barbara Jonchère, Eve Maubec, Agnès Carlotti, Eduardo Marinho, Benoit Couturaud, Martine Peter, Xavier Sastre-Garau, Marie-Françoise Avril, Nicolas Dupin, Flore Rozenberg

**Affiliations:** 1 Université Paris Descartes, EA1833, Paris, France; 2 Assistance Publique - Hôpitaux de Paris, Hôpital Cochin, Service de Virologie, Paris, France; 3 Assistance Publique - Hôpitaux de Paris, Hôpital Bichat, Service de Dermatologie, Paris, France; 4 Assistance Publique - Hôpitaux de Paris, Hôpital Cochin, Service d'Anatomopathologie, Paris, France; 5 Assistance Publique - Hôpitaux de Paris, Hôpital Bichat, Service d'Anatomopathologie, Paris, France; 6 Institut Curie, Service de Chirurgie, Paris, France; 7 Institut Curie, Laboratoire d'Anatomopathologie, Paris, France; 8 Assistance Publique - Hôpitaux de Paris, Hôpital Cochin, Service de Dermatologie, Paris, France; Fred Hutchinson Cancer Research Center, United States of America

## Abstract

Merkel Cell Polyomavirus (MCPyV) is associated with Merkel Cell carcinoma (MCC), a rare, aggressive skin cancer with neuroendocrine features. The causal role of MCPyV is highly suggested by monoclonal integration of its genome and expression of the viral large T (LT) antigen in MCC cells. We investigated and characterized MCPyV molecular features in MCC, respiratory, urine and blood samples from 33 patients by quantitative PCR, sequencing and detection of integrated viral DNA. We examined associations between either MCPyV viral load in primary MCC or MCPyV DNAemia and survival. Results were interpreted with respect to the viral molecular signature in each compartment. Patients with MCC containing more than 1 viral genome copy per cell had a longer period in complete remission than patients with less than 1 copy per cell (34 vs 10 months, *P* = 0.037). Peripheral blood mononuclear cells (PBMC) contained MCPyV more frequently in patients sampled with disease than in patients in complete remission (60% vs 11%, *P* = 0.00083). Moreover, the detection of MCPyV in at least one PBMC sample during follow-up was associated with a shorter overall survival (*P* = 0.003). Sequencing of viral DNA from MCC and non MCC samples characterized common single nucleotide polymorphisms defining 8 patient specific strains. However, specific molecular signatures truncating MCPyV LT were observed in 8/12 MCC cases but not in respiratory and urinary samples from 15 patients. New integration sites were identified in 4 MCC cases. Finally, mutated-integrated forms of MCPyV were detected in PBMC of two patients with disseminated MCC disease, indicating circulation of metastatic cells. We conclude that MCPyV molecular features in primary MCC tumour and PBMC may help to predict the course of the disease.

## Author Summary

Merkel cell polyomavirus (MCPyV) is a recently discovered virus highly associated with a rare skin cancer, Merkel cell carcinoma (MCC). The causal role of MCPyV in cancer is suggested by integration of viral sequences into the cell genome and by a specific molecular signature. We looked for and compared molecular species of MCPyV in tumour and non tumour samples of 33 MCC patients. We showed that a tumour viral load greater than 1 copy per cell was associated with a better outcome, and that detection of the virus in blood but not in urine correlated with a shorter overall survival. A tumour–specific molecular signature was found in the blood of two patients with metastatic disease, but did not occur in their respiratory nor urine samples. We propose that molecular analysis of MCPyV in tumour and blood be used as a biomarker of infection and cancer progression in MCC patients.

## Introduction

Polyomaviruses are small, non enveloped double stranded DNA viruses which infect many species with a restricted host range. The initial discovery of Murine polyomavirus (MPyV) and Simian vacuolating 40 (SV40) was closely linked to the demonstration of their experimental tumorigenic properties [1]. Infections by the human polyomaviruses BK (BKPyV), JC (JCPyV), and the recently identified KI (KIPyV) and WU (WUPyV) are highly prevalent in most populations [2], [3]. Polyomaviruses persist latently in the host and may reactivate, causing disease in the immunocompromised [4], [5], but have not been firmly associated with cancer in humans [6]. Therefore, the discovery in a rare but aggressive skin cancer, Merkel Cell Carcinoma (MCC), of a fifth human Polyomavirus, named Merkel Cell Polyomavirus (MCPyV) has raised new interest in the oncogenic potential of human Polyomaviruses [7]. MCPyV DNA was shown to be monoclonally integrated into most MCC, and tumour cells were found to express the major viral oncoprotein, large T antigen (LT) [8]. Remarkably, MCPyV present in MCC tissue exhibited a molecular signature, consisting of mutations which truncate LT and suppress its helicase domain required for viral replication [9]. These features are similar to molecular defects observed in MPyV [10] and bring strong evidence for a causative role of the virus in MCC.

MCC is a carcinoma of neuroendocrine cells which affects principally elderly and immunocompromised patients. The close association between MCPyV and MCC has been confirmed in several case series worldwide [11], [12], [13], [14], [15], [16], [17], [18], [19], [20], [21], [22]. However, MCPyV has been detected in normal skin [23], benign skin lesions [24] and non MCC skin cancer [25], and also found in various tissues such as the oral cavity [26], gastrointestinal and urogenital tracts [23], tonsils [27], respiratory tract [28], blood [9], and rarely in non skin cancers [19], [25], [29], [30]. Seroepidemiologic studies have revealed that most healthy individuals are infected with MCPyV, as with other human Polyomaviruses [31], [32], [33], [34], [35]. Therefore, numerous questions related to the persistence and replication of the virus in the host and mechanisms of oncogenesis remain unanswered. We investigated whether MCC patients' clinical data were related to the presence of MCPyV and specific molecular features in tumour and non tumour tissues. In a cohort of 33 MCC patients, we collected MCC and non MCC samples, and compared the frequencies of MCPyV DNA detection, viral load and nucleotide sequences. We looked for integration of MCPyV in MCC cells and for the presence of integrated/mutated forms of MCPyV in non tumour samples.

## Materials and Methods

### Ethics statement

This study was conducted according to the principles expressed in the Declaration of Helsinki. According to French regulation, information was delivered to the patients on research performed on biological samples, and written informed consent was obtained for participation in the study, which was approved by the institutional review board of the Comité de protection des personnes Ile de France 3.

### Patients and samples

All patients with Merkel cell carcinoma who attended the Dermatology Departments of Cochin and Bichat hospitals from March 2008 to April 2010 were prospectively included in the study. Histopathologic confirmation of MCC diagnosis relied on tumour morphology consistent with MCC on hematoxylin-eosin-stained tissue sections, paranuclear dot immunostaining pattern for CK20 or positive immunostaining for synaptophysin and chromogranin A. Clinical data were retrieved from hospital case records, and included sex, age at diagnosis, site and size of primary MCC, and stage of the disease at diagnosis, according to Allen's classification [36] based on primary tumour diameter (<2 cm  = I, >2 cm  =  II) and the presence of metastasis (regional lymph node metastasis  =  III, distant  =  IV). Samples from primary and/or metastatic MCC lesions were recovered and included retrospectively retrieved formalin-fixed paraffin-embedded (FFPE) sections and/or fresh-frozen specimens for patients newly diagnosed or who relapsed. FFPE sections from non MCC cancer tissue were retrieved if possible. In addition, blood and/or nasal swab and/or urine were sampled in patients at inclusion and follow-up visits when possible, and disease stage and status were recorded. Status was defined according to the presence or absence of tumour (primary and/or metastatic) as alive with disease (AWD) or in complete remission (CR) respectively. Date and cause of death were recorded.

### Detection and quantification of MCPyV sequences in MCC and non MCC samples

For FFPE samples, two consecutive 10 µ-thick sections were retrieved. Frozen fragments were cut and mechanically dissociated. DNA was extracted using the QIAmp DNA tissue extraction kit (Qiagen) according to the manufacturer instructions. Nasal swabs discharged in 200 µl sterile saline buffer and urine were immediately frozen and kept at –20°C until analysis. PBMC were obtained by Ficollpaque centrifugation (Eurobio) of 3 mL EDTA blood, suspended in 200 µl of PBS, immediately frozen and kept at –20°C until analysis. DNA was extracted using the QIAmp blood DNA extraction kit (Qiagen). For tissue material, lysis was extended to 24 h. DNA was eluted in 200 µl elution buffer (Macheret), and concentration was measured by UV-spectrometry (NanoDrop Technology, Willmington).

To detect MCPyV sequences, a sensitive real time PCR assay was designed using primers which encompass a 91 bp fragment of the LT oncoprotein gene, located upstream of the Rb-binding encoding sequence (Table S1) [7]. Amplification was performed on 100 ng DNA with 300 nM each primer and 100 nM probe in 50 µl Taqman Master Mix (Applied Biosystems, Courtaboeuf). After a 10 min denaturation step at 95°C, cycling conditions consisted of 50 cycles of 15 sec at 95°C and 1 min at 60°C on an ABI 7500 platform (Applied Biosystems). To check for carryover between the samples, mock samples were included in each series. Confirmation LT3 and VP1 PCR assays were performed as described [7]. Absolute quantification of MCPyV viral load in patients' samples was obtained by establishing a calibration curve with ten-fold serial dilutions (from 10 to 10^6^ copies) of known concentrations of plasmid MCCIC13 which contains 1 copy of the MCPyV genome inserted in the pCR-XL-TOPO vector [18]. To quantify MCPyV DNA in copy number per cell in MCC and non MCC cancer samples, the housekeeping apolipoprotein B gene (hapb) [37] was amplified by real time PCR and the delta-delta Ct method was used.

### Detection of BKPyV and JCPyV sequences

To detect BKPyV and JCPyV sequences, real time PCR assays were designed to amplify a 79 and a 110 bp fragment of the LT antigen coding sequence in BKPyV and JCPyV genome respectively (Table S1). Amplification was performed using the same cycling and control conditions as above.

### Analysis of MCPyV integration site

To characterize integration of MCPyV, we used the DIPS-PCR technique which amplifies junctions between the host and MCPyV genomes as previously described [18], [38]. Briefly, DNA extracted from MCC tumours was digested overnight with Taq I (Invitrogen), an enzyme that does not cleave the MCV350 sequence (GenBank accession number EU375803), and ligated to adapters. Ligated fragments were subjected to 40 cycles of linear amplification with forward primers sequentially designed along the MCPyV LT gene (Table S1), followed by a 30 cycle exponential amplification using internal primers coupled to a reverse adapter-specific primer. PCR products were visualized and cut from agarose gel, purified with QIAmp gel extraction kit (Qiagen), and directly sequenced using Big-Dye terminator DNA-Sequencing technology (Applied Biosystems).

### PCR detection of integrated and non integrated MCPyV genome

To discriminate integrated versus non integrated forms of the MCPyV genome in patients' samples, we amplified short fragments bracketing each of the characterized integration sites. DNA (100 ng) was subjected to PCR using a forward MCPyV-specific primer located 100 to 300 bp upstream of the integration site, and a reverse downstream primer, specific either to the human integration locus or to the MCPyV non-integrated genome (Table S1). PCR was performed in 50 µl Ampli Taq Gold Master Mix (Applied Biosystems) for 50 cycles with conditions specific to each primer set.

### Sequencing

Overlapping fragments of the MCPyV LT encoding gene were amplified using four primer pairs in order to cover the whole second exon of LT (base pair number 861 to 3080 according to the MCV 350 genome (Table S1). PCR was performed on 100 ng DNA by using HotStartTaq Master Mix kit (Qiagen) containing 0.5 µM primers in a final volume of 50 µL. PCR products were then directly sequenced as above.

### Statistical methods

The cumulative rates of survival in complete remission relative to MCPyV load in primary MCC (<1 or ≥1 copy per cell), and overall survival relative to the presence or absence of MCPyV DNA in PBMC were estimated by the Kaplan-Meier method. Patients with follow-up under 3 months were excluded from analysis. Survival in complete remission was calculated from the date of diagnosis to the date of first tumour recurrence in patients AWD or died of disease (DOD) at last follow-up. Patients in complete remission were censored at their last follow-up visit. Analyses were performed using the XLStat software (Addinsoft, Paris, France). Multivariable analysis of survival in complete remission was done by using a stepwise Cox proportional hazards model that used forward covariate entry to the model. The proportions of patients either CR or AWD, or at stages I or II or III versus IV who had either a MCPyV negative or MCPyV positive PBMC sample were compared using the exact Fischer test. All *P* values were two-sided, and *P* values less than.05 were considered statistically significant.

## Results

### Clinical findings

Thirty nine patients with MCC attended the Dermatology Departments of Bichat and Cochin hospitals. Six patients without retrieved MCC material were excluded from the study. The remaining 33 patients included 14 males and 19 females (sex ratio  = 0.6). Their median age at diagnosis was 77 years (range 39–88). Four patients were immunocompromised, because of corticoid therapy for rheumatoid arthritis, hepatic transplantation, lymphopenia and recurring hairy cell leukaemia. Thirteen (39%) patients had a history of cancer other than MCC (non MCC skin cancer and/or non skin cancer) (Table S2). Primary MCC was localized to the limbs, head, and trunk in 21 (64%), 11 (33%) and 1 (3%) cases respectively. MCC median diameter was 25 mm (range 7–70 mm). At diagnosis, patients were at Allen's stages I, II, III and IV in 9 (27%), 16 (48%), 7 (21%) and 1 (3%) cases respectively [36]. The median delays from diagnosis until inclusion and last follow-up were 7 months (up to 112 months) and 16 months (up to 134 months) respectively. At last follow-up, 18 (54%) patients were in CR, 8 (24%) patients were AWD and 7 (21%) patients had died of disease (DOD) (Table 1).

**Table 1:** Clinical data of MCC patients.

Case	Sex/Age^a^	Primary MCC data			Inclusion visit			Last follow-up visit		
		Size (mm)	Site	Stage b	Delay c	Status d	Stage b	Delay c	Status d	Stage b
1	F/74	40	Thigh	II	10	AWD	III	16	DOD	IV
2 e	M/81	10	Ankle	III	30	AWD	III	47	AWD	III
3 e	M/78	22	Wrist	II	15	AWD	IV	33	DOD	IV
4	F/73	55	Cheek	III	1	AWD	III	6	CR	III
5	F/61	7	Cheek	I	25	CR	I	26	CR	I
6	M/88	25	Leg	II	0	AWD	II	0	AWD	II
7 e	M/84	45	Ear	II	3	AWD	IV	4	DOD	IV
8	F/67	10	Cheek	I	7	CR	I	9	CR	I
9 f	M/82	10	Mandible	I	1	CR	I	8	AWD	III
10	F/82	21	Leg	III	23	CR	III	29	CR	III
11	F/77	10	Wrist	I	0	CR	I	0	AWD	I
12	M/55	50	Forearm	III	5	AWD	III	6	AWD	III
13	M/71	30	Arm	III	24	CR	III	24	CR	IV
14 e,f	M/63	15	Nipple	III	0	AWD	III	31	CR	III
15	F/86	20	Leg	IV	1	AWD	IV	8	AWD	IV
16 f	F/77	25	Knee	II	7	AWD	IV	9	DOD	IV
17	F/65	8	Arm	I	1	AWD	I	4	CR	I
18	M/68	20	Thigh	II	41	AWD	IV	41	DOD	IV
19	F/66	30	Thigh	II	48	CR	II	71	CR	II
20	F/85	25	Cheek	II	1	AWD	II	23	CR	II
21	M/60	70	Arm	III	4	AWD	III	5	CR	III
22	F/81	10	Cheek	I	35	CR	I	41	CR	I
23 e	M/85	40	Buttock	II	21	AWD	IV	21	DOD	IV
24	F/71	30	Cheek	II	37	CR	II	37	CR	II
25	F/63	10	Cheek	I	0	CR	I	16	CR	II
26	F/39	30	Arm	II	112	CR	II	134	CR	III
27	F/81	15	Thigh	I	34	CR	I	53	CR	I
28	F/58	30	Leg	II	1	AWD	II	10	CR	II
29	F/77	18	Ear	I	1	AWD	I	10	CR	I
30	M/80	40	Vertex	II	1	CR	II	15	AWD	III
31 e,f	M/84	27	Thigh	II	14	AWD	IV	17	DOD	IV
32	M/60	45	Arm	II	13	AWD	III	19	AWD	IV
33	F/81	30	Arm	II	1	AWD	II	3	CR	II

a Age at diagnosis.

b Disease stage according to Allen's classification.

c Delay in months from MCC diagnosis.

d Disease status relative to presence (alive with disease or AWD) or absence (complete remission or CR) of tumour and death (died of disease or DOD).

e Patients partly previously reported [18].

f Immunocompromised patients.

### MCPyV DNA viral load in MCC samples and outcome

**Figure 1:**
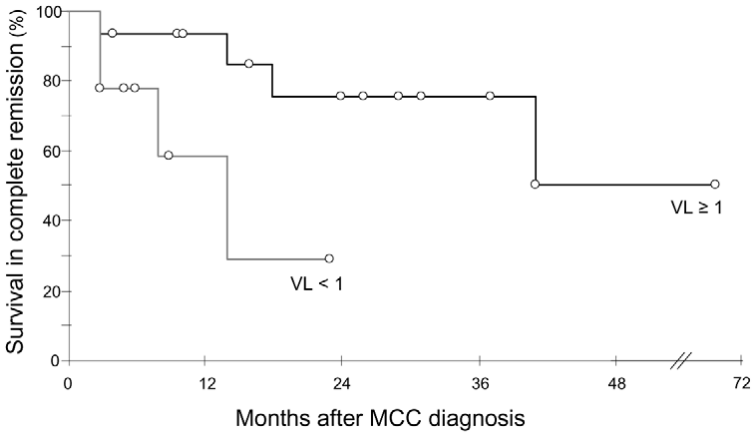
Kaplan Meier analysis of survival in complete remission relative to primary tumour MCPyV load. Patients with ≥1 genome copy per cell (n = 15, black curve) had longer survival in complete remission than patients with <1 genome copy per cell (n = 9, grey curve). VL : viral load. Open circles indicate patients censored at last follow-up visit.

We analyzed 43 MCC samples consisting of 26 primary MCC (15 fresh-frozen and 11 formalin fixed paraffin-embedded (FFPE) specimens), 14 skin metastasis (12 fresh-frozen and 2 FFPE specimens) and 3 fresh-frozen regional lymph node metastasis samples. Viral DNA was detected in 41/43 samples from 31/33 patients, with a median viral load quantified in 37 samples of 3 copies per cell (range 3.10^−3^ to 3.10^3^). Negative results observed in one FFPE section and one fresh frozen sample from primary MCC were confirmed using the previously described LT3 and VP1 PCR assays [7]. In twenty four patients with follow up greater than three months, viral load in the primary tumour was analyzed with respect to survival. Median survival in complete remission was longer in patients who had ≥1 copy per cell (n = 15), than in patients who had no detectable viral DNA (n = 2) or <1 copy per cell (n = 7) (34 months, 95% CI  = 26 to 42 vs 10 months, 95% CI  = 7 to 14, Kaplan Meier log-rank P = 0.037) (Figure 1). Among clinical parameters analyzed (sex, age, limb site and size of primary tumour, and presence or absence of lymph node metastases at diagnosis), which didn't differ in the two groups, only female sex was associated with a better outcome. Adjusted for sex, the relative hazard for survival in complete remission was 4.8 (95% confidence interval 0.90–26, P = 0.066) with primary tumour containing more than 1 copy per cell.

### MCPyV DNA in non tumour samples of MCC patients, disease stage and outcome

We then asked if, in MCC patients, MCPyV was restricted to MCC tissue. Viral DNA was detected in 27/28 (96%) nasal swabs from 21/21 patients, with a median load of 3.10^3^ copies per sample (range 5–2.10^6^). MCPyV DNA was also found in 22/38 (58%) urine samples from 18/28 (64%) MCC patients, with a median load of 6.10^2^ copies/ml (range 100–4.10^5^). In addition, MCPyV was amplified from 20/49 (41%) PBMC samples from 15/30 (50%) patients with a median load of 10^2^ copies per ml whole blood (range 10–5.10^4^). MCPyV DNA was detected in 1/6 FFPE non MCC cancer samples from 5 patients (Table S2). We wondered whether the high rate of detection of MCPyV was common to other human Polyomaviruses. BKPyV and JCPyV DNA were amplified from 9% each of nasal swabs, 31% and 7% of urine samples, and 3% and 6% of PBMC respectively.

**Figure 2:**
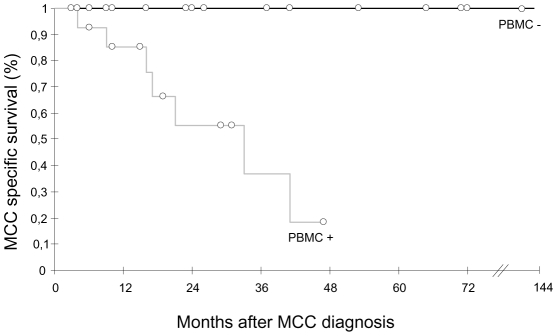
Kaplan Meier analysis of overall survival relative to MCPyV detection in PBMC. Patients who had all MCPyV-negative PBMC (n = 14, black curve) had a longer survival than patients who had at least one MCPyV-positive PBMC (n = 14, grey curve). Open circles indicate patients censored at last follow-up visit.

Since MCPyV DNA was detected infrequently in urine and in the PBMC of about half of the patients, we asked whether MCPyV DNAuria and/or DNAemia were linked to the stage and/or evolution of the disease. No correlation between MCPyV detection in urine was found with any of these parameters. In contrast, detection of MCPyV in PBMC was more frequent in patients sampled alive with disease (AWD) than in patients in complete remission (CR) (18/30 or 60% versus 2/19 or 11%, *P* = 0.00083). Moreover, among AWD patients, MCPyV tended to be more frequently detected in patients with distant metastasis than at less advanced stages of the disease (6/6 MCPyV positive PBMC when sampled at stage IV versus 12/24 or 50% when sampled at stages I, II or III, *P* = 0.057). In twenty eight patients with follow up greater than three months, the detection of MCPyV in PBMC was associated with poorer outcome, since patients with at least one positive sample had a median survival of 28 months (95% IC  = 19 to 36) whereas all patients with no detectable MCPyV survived after a median follow-up of 71 months (Kaplan Meier log-rank *P* = 0.003) (Figure 2). All clinical parameters analyzed were comparable in the PBMC-positive and PBMC-negative groups except sex, since significantly more male patients had MCPyV-positive PBMC (P<0.009). Among these parameters, only primary tumour size above 2 cm was associated with higher risk of death. We then looked at PBMC results according to viral load in primary MCC. Among 15 patients with ≥1 copy per cell, 4/4 patients who relapsed had positive PBMC, compared with 3/11 disease-free patients. Among 9 patients with <1 copy per cell, 8 had a PBMC sample tested. Two of four patients who relapsed tested positive while two with MCPyV-negative tumours tested negative. The four patients who were disease free at last follow-up had negative PBMC.

### MCPyV integration in MCC samples

Using the DIPS-PCR method, we demonstrated the integration of MCPyV DNA in six MCC cases. We first confirmed viral integration in metastatic tissue from two patients, and found virus-host genome junctions identical to those previously reported in their primary tumours [18]. In three new cases, integration of MCPyV was found to interrupt the second exon of LT downstream from the Rb binding coding sequence, whereas it interrupted the 3′ end of the VP1 gene in another one. Integration was located on four distinct chromosomal loci, next to or into known human genes (Table 2). Two of them, PARVA and DENND1A genes, encode proteins involved in cell junctions and in formation of clathrin coated vesicles or cell adhesion and cytoskeleton organization respectively. A third gene, TEAD1, encodes a transcriptional activator reported to be used by the SV40 enhancer to activate expression of the early T oncoprotein gene [39]. Finally, in one case, integration resulted in fusion of the MCPyV LT gene and successive truncated fragments of the seventh and the tenth introns of the GMDS gene, two regions separated by approximately 200 kb in the human genome, demonstrating that large rearrangements occurred.

**Table 2:** Identification of four novel integration sites of MCPyV DNA in MCC genome.

Case	MCPyV 3′ breakpoints a	Chromosome insertion site	Putative target human gene	
			Gene	Distance
4	2166 (LT)	15q14	ATP binding domain 4 isoform 2 (ATPBD4)Hypothetical protein XP_002343377 (mRNA similar to cytochrome c oxidase, subunit VIc)	353 kbp (5′)672 kbp (3′)
16	3337 (VP1)	9q33	DENN/MADD domain containing 1A isoform 1 (DENND1A)mir601	2nd intron600 bp (3′)
20	2981 (LT)	11p15	Parvin alpha (PARVA)TEA domain family member 1 (TEAD1) or SV40 transcriptional enhancer factor	8 kbp (5′)228 kbp (3′)
29	2594 (LT)	6p24	GDP-mannose 4,6 deshydratase (GMDS)	7th/10th introns

3′ virus-host junction was characterized by DIPS PCR.

a Base-pair designations correspond to the prototype MCV 350 (accession number NC_010277). LT  =  Large T, VP1  =  Major viral capsid.

### Single nucleotide polymorphisms define patients' strains

**Figure 3:**
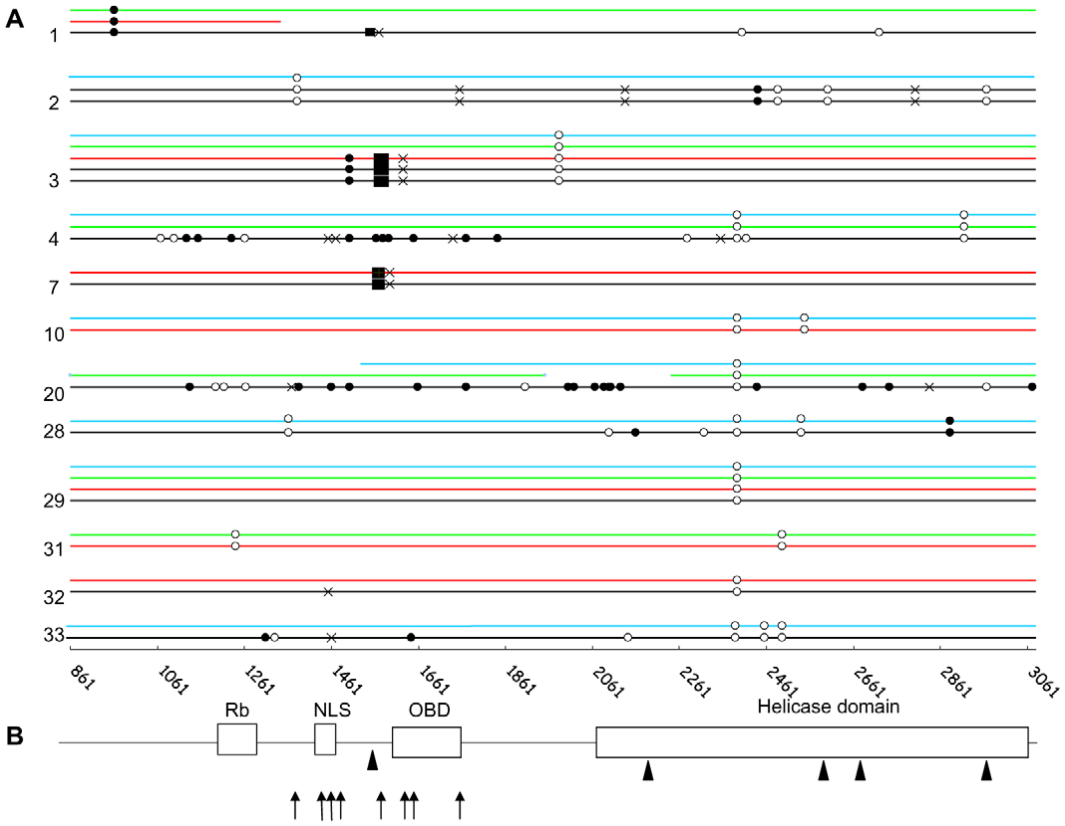
Characterization of MCC patients' strain and tumour signature in MCPyV LT. **A**. Direct PCR sequencing of the second exon of MCPyV LT in MCC tumours. Twelve distinct representative cases (case number indicated on the left) are represented. Sequences from MCC (black), nasal swab (blue), urine (green) and PBMC (red) tissues reveal single nucleotide polymorphisms (SNPs), and tumour-specific mutations: open circle, synonymous; filled circle, non synonymous; black cross, premature stop codon; filled box, deletions. In cases 1 and 20, weak viral load in PBMC and nasal swab respectively prevented full length amplification of LT. Base pair designation as in Table 2. **B**. Diagram representation of main functional domains encoded by the second exon of LT (Rb  =  Rb binding domain, NLS  =  nuclear localization signal, OBD  =  origin binding domain and helicase domain). Arrows indicate premature truncating mutations determined by direct sequencing and arrowheads indicate the site of integrated viral DNA determined by 3′ DIPS-PCR. GenBank accession number: HM587427-HM587448.

We sequenced the whole second exon of LT gene in MCC and non MCC tissues. Fifty-two sequences from 26 patients displayed >99% homology with prototypes MCC350, MCC339, MKL-1 and TKS published sequences [7], [9]. To characterize strain-specific and/or tumour-specific markers, a local consensus reference was constructed by alignment of all sequences, and variations were indicated as silent, non synonymous or stop mutations, deletions or insertions. The total number of silent and non synonymous mutations in MCC and non MCC samples with respect to the consensus differed by three and sixteen fold respectively. Single nucleotide polymorphisms (SNPs) characterized strain specific signatures in eight patients, and a common single silent mutation was identified in three other patients (Figure 3A).

### MCC molecular signature differentiates tumour and non tumour samples

Fourteen MCC sequences (13 complete and 1 partial) were obtained from 12 patients. In two patients, we verified that sequences from distinct metastasis exhibited 100% homology. Mutations which truncate LT were characterized in nine cases, consisting in stop mutations (5 cases), or deletions causing frameshifts which generate stop codons (3 cases) (Figure 3A). In the remaining case, a 250 bp insertion of the third intron of the DENND1A gene was lying inside LT, upstream the VP1-host genome junction identified in the second intron of the same human gene. Overall, mutations preserved the Rb fixation domain but inactivated the helicase domain of the oncoprotein (Figure 3B). In one additional patient, we failed to amplify the 3′ end 600 last bp of LT, despite a high viral load and repeated attempts using different sets of primers, suggesting a truncation of this sequence. Finally, in the last two cases, full length sequences were obtained and encoded a non truncated protein. Interestingly, in 5 MCC cases where the DIPS-PCR characterized integrated-truncated LT, a complete LT sequence was amplified downstream the truncation site, suggesting the coexistence of integrated concatemers or latent episomes of the MCPyV genome and truncated-integrated viral sequences.

Then, we analyzed 16 nasal and 8 urine sequences. All sequences were complete except three obtained from weak positive samples, probably reflecting sensitivity limits of the method used rather than truncations. Two sequential nasal sequences from the same case showed 100% homology. All full-length sequences were wild-type. None of the 9 non MCC samples from 7 patients who displayed a truncated LT in MCC harboured the tumour-specific molecular signature (Figure 1A). These results suggest that nasal swabs or urine are likely to contain MCPyV DNA from either excreted or episomal virus.

PBMC sequences were obtained in 14 cases, including partial sequences obtained from 7 weak positive samples. Complete PBMC and MCC sequences from 4 patients were compared (Figure 1A). In one case, both PBMC and MCC sequences encoded intact LT antigen open reading frame (ORF). In another case, the premature stop codon observed in MCC LT was absent from MCPyV recovered from PBMC. In contrast, in two other cases, the specific MCC signatures (a 5 and a 25 bp deletion respectively) were recovered from the two patients' PBMC samples. Notably, in one of these patients, sequences from nasal swabs and urine were also analyzed and didn't harbour the tumour signature. Since the two patients presented disseminated metastatic lesions at the time of sampling and were both DOD at the end of the study, we assume that the presence of mutated MCPyV DNA in PBMC reflected circulation of metastatic MCC cells. To further confirm this last hypothesis, we amplified a portion of the LT gene bracketing the predicted integration site and the viral-host junction sequence in tumour and non tumour samples of five patients. Virus-host junction sequences were amplified from MCC tissue in all cases, from PBMC in two cases, but from neither urine nor nasal swabs. Both integrated and non integrated products were amplified from MCC samples, further suggesting the coexistence of integrated-truncated viral sequences and integrated concatemers or episomes in MCC samples. Altogether, these results suggest that MCPyV sequences recovered at peripheral urine and respiratory sites merely correspond to free excreted virus or episomal DNA, whereas the presence in the patients' PBMC of tumour-like sequences argued for the presence of circulating tumour cells.

## Discussion

Since the discovery by Feng et al who identified MCPyV in 6/8 MCC [7], several large studies have demonstrated that MCPyV is associated with most cases of MCC except in Australia [11], [12], [13], [17], [19], [22], [24], [26], [33]. We detected MCPyV in MCC from 31/33 patients. Negative results from FFPE specimens could be due to poor conservation of DNA. Only one fresh frozen tumour tested negative. We estimated MCC median viral load at 3 copies per cell, with a 6 log variation between samples, consistent with other reports [13], [18], [22], [40], [41], [42], [43]. Variations may be due to tissue quality, the proportion of non tumour cells in samples, or mutations in the target viral sequence. However, variations in rates of LT expressing MCC cells were also reported [8], [43]. The fact that some MCC cases do not contain MCPyV DNA nor express LT suggests that MCC is a heterogeneous disease with at least two etiologies, despite a lack of phenotypic markers able to distinguish between MCPyV positive and MCPyV negative cases [8], [44]. Interestingly, patients with MCC containing at least 1 copy of viral genome per cell had better outcome than patients with lower values of MCPyV. Although the low number of patients studied impairs definite conclusions, it is striking that two previous studies also reported poorer survival rate in patients with the lowest viral DNA load and LT expression in MCC [22], [40]. Although the mechanisms of MCC pathogenesis are unknown, variations in MCPyV load and in patients outcome further argue for heterogeneity and variable implication of the virus in the disease [11], [12], [16], [21], [45], as previously described in HPV related and unrelated carcinoma [46]. Several observations support the causal role of MCPyV in most MCC. In particular, cell transformation by MCPyV was shown to depend on LT, as in other polyomaviruses-induced oncogenesis [1]. First, MCPyV LT is able to bind and sequester the tumor-suppressor protein Rb through a conserved LxCxE motif [9]. Second, the transformed phenotype of MCPyV-positive MCC cell lines depends on LT expression, since cells undergo growth arrest and/or death upon LT silencing [47]. However, in two models of adenovirus and polyomavirus-induced oncogenesis, the dependence of transformed cells on viral oncoproteins was reversed upon time, since cells conserved an oncogenic phenotype while viral expression was shut-down in one case and viral sequences were lost in the other [48], [49]. These findings suggest that transformed cells acquire subsequent genetic alterations which circumvent their need for a continued expression of viral oncoproteins. Therefore, more cellular genetic alterations may be necessary in virus-unrelated than in virus-related oncogenesis. In this respect, the number of chromosomal alterations and amplifications was significantly higher in HPV-unrelated than in HPV-related carcinomas, and correlated with unfavourable prognosis [46]. Recurrent genomic changes have been described in MCC [16], [18], but their link with MCPyV has not yet been extensively investigated.

Monoclonal integration of MCPyV is viewed as a key element in oncogenesis. We identified in metastatic lesions from two patients the same virus-host genome integration characteristics previously described in their primary tumours, sustaining the hypothesis that viral integration constitutes an early event in MCC oncogenesis [7], [18]. We also showed the integration of truncated LT in four cases, and in one of these this led to a complex rearrangement between LT, VP1 and the target human gene. All chromosomal integration sites identified so far differ from each other [7], [18]. We are currently verifying whether expression of putative target human genes, located in the vicinity or at the site of integration, is modified in tumour cells, notably TEAD1 which was reported to be used by another Polyomavirus, SV40, as a transcriptional enhancer factor. In addition, MCC LT sequences revealed various point or frameshift mutations which preserve the Rb binding domain but truncate the oncoprotein before the helicase domain, as in the tumour-specific molecular signatures previously described [9], [50]. Such truncations preserve the transformation ability of LT through Rb sequestration, but prevent viral DNA replication. A similar loss of full-length LT has been observed *in vitro* and *in vivo* in models of SV40 and MPyV-induced carcinogenesis [51], [52] In addition, replication-defective polyomaviruses with loss of LT binding to the origin of replication showed enhanced transforming properties [53]. Our results extend previous observations and reinforce the hypothesis that acquisition of mutations within LT is a common feature and may be a prerequisite for carcinogenesis induced by polyomaviruses. However, in three cases of this series and in two previously reported cases, mutations truncated LT upstream an identified nuclear localization signal, which could prevent nuclear expression of the protein [9]. Lastly, mutations in LT were not observed in all cases in this nor in other studies [43], [54]. We can't exclude that these cases display mutations at other sites critical for MCPyV replication. A point mutation in a pentanucleotide sequence of the replication origin was observed in a MCC strain and prevented replication [55]. Finally, the fact that the full length second exon of LT was sequenced in five MCC samples although integration interrupted LT suggests that, as previously observed with Southern Blot analysis [9], truncated/integrated and probably whole genomic copies of MCPyV coexist in tumour cells, as confirmed by PCR assay which discriminates integrated versus non integrated MCPyV genomes.

The lifecycle of MCPyV in the host is unknown. Serological studies showed that infection is common in the general population and occurs before the third decade [33], long before development of MCC. Routes of transmission and sites of excretion are not completely known. We showed presence of MCPyV in the respiratory tract of most MCC patients, in serial samples drawn at a several-month interval, in contrasts with low detection rate (below 17%) in non MCC patients reported in the literature and observed with our own detection method (data not shown) [4], [27], [28], [50], [56], [57], [58]. MCPyV DNA excretion in urine, which was previously reported in one MCC case [59], was observed in almost half of patients, above rates (below 25%) reported in control subjects [23], [26]. Comparative LT sequencing from MCC and non MCC samples revealed strain-specific SNPs. Whereas most MCC sequences displayed tumour-specific molecular signatures, all nasal swabs and urine sequences were wild-type, suggesting that the latter correspond to excreted or episomal virus, whereas the former belong to integrated genomes. Thus, high rates of MCPyV excretion both in the respiratory tract and urine may be a hallmark of MCC patients. Urine excretion of BKPyV or JCPyV is frequent in immune competent subjects and increases with age, during pregnancy or immune suppression [60], [61]. Since excretion rates of BKPyV and JCPyV were comparable in MCC patients to rates of non MCC patients [61], [62], we hypothesize that patients present a specific failure to control latency of MCPyV but not all Polyomaviruses. This hypothesis is supported by the fact that high levels of antibodies directed towards the major viral capsid protein VP1 of MCPyV but not other human Polyomaviruses were more frequently observed in MCC patients than in the general population [31], [34]. Indeed, in the case of BKPyV and JCPyV infection, reactivation and active shedding were positively correlated with serum antibody responses to VP1 [63], [64].

Lastly, our results show evidence of high rates of MCPyV DNA in MCC patients' PBMC, in contrast with low rates (0–8%) reported in the serum, whole blood or PBMC of non MCC subjects [17], [19], [27], [29], [50]. Moreover, MCPyV DNA detection in PBMC was significantly correlated with the disease stage and outcome since patients with at least one PBMC positive sample had shorter survival in remission that patients in whom MCPyV had not been detected in any PBMC sample. In one patient, MCPyV recovered from PBMC had a wild-type genotype whereas the viral genome recovered from MCC had a LT truncating mutation. We hypothesize that MCPyV DNAemia may correspond to active viral replication following reactivation, as observed with other human polyomaviruses [2], [61]. Indeed, MCPyV DNA detection was reported in activated circulating monocytes of one MCC patient and one control [59]. In two patients in our study however, viral sequences recovered from PBMC displayed the patient's MCC-specific molecular signature. As both patients were sampled at a metastatic stage and subsequently died of their disease, we believe that PBMC viral DNA revealed metastatic circulating cells, since MCC cells were previously identified in the peripheral blood of one MCC patient [65].

Altogether, our results provide new insights in the life cycle of MCPyV during MCC pathogenesis. The low number of cases studied might weaken the statistical power of our results. However, we suggest that quantitative and qualitative molecular analysis of MCPyV in tumour and non tumour sites of MCC patients may be a useful tool to characterize their disease stage and manage their follow-up. We are currently designing a prospective study to confirm these results in large series of patients.

## Supporting Information

Table S1 Primers and probes used for PCR, DIPS-PCR and sequencing.
(0.03 MB XLS)

Table S2 Non MCC cancer data of MCC patients. FFPE samples were tested for MCPyV DNA. Case number as in Table 1. BCC, basal cell carcinoma; SCC, squamous cell carcinoma. NA, non available.
(0.02 MB XLS)
